# A Paper-Based Colorimetric Aptasensor for the Detection of Gentamicin

**DOI:** 10.3390/bios11020029

**Published:** 2021-01-21

**Authors:** Saipriya Ramalingam, Christopher M. Collier, Ashutosh Singh

**Affiliations:** School of Engineering, University of Guelph, Guelph, ON N1G 2W1, Canada; sramalin@uoguelph.ca (S.R.); ccollier@uoguelph.ca (C.M.C.)

**Keywords:** gentamicin, aptamer, gold nanoparticles, colorimetric biosensor, paper microfluidics, aptamer

## Abstract

Antibiotics are classes of antimicrobial substances that are administered widely in the field of veterinary science to promote animal health and feed efficiency. Cattle-administered antibiotics hold a risk of passing active residues to milk, during the milking process. This becomes a public health concern as these residues can cause severe allergic reactions to sensitive groups and considerable economic losses to the farmer. Hence, to ensure that the produced milk is safe to consume and adheres to permissible limits, an on-farm quick and reliable test is essential. This study illustrates the design and development of a microfluidic paper biosensor as a proof-of-concept detection system for gentamicin in milk. Localized surface plasmon resonance (LSPR) properties of gold nanoparticles have been explored to provide the user a visual feedback on the test, which was also corroborated by RGB analysis performed using Image J. The assay involves the use of a short stretch of single stranded DNA, called aptamer, which is very specific to the gentamicin present in the milk sample. The camera-based LOD for the fabricated paper device for milk samples spiked with gentamicin was calculated to be 300 nM, with a reaction time of 2 min.

## 1. Introduction

Food-producing animals, such as dairy cows, have been administered antibiotics as part of disease control and regular well-being since the early 1930s. Antibiotics, also known as antibacterials, are synthetic/semi-synthetic chemical compounds that retard or eliminate the growth of bacteria. Statistics from the Centre for Disease Control and Prevention (CDC) as of 2020 reveal that at least 2.8 million people have developed significant drug resistance, of which 35,000 lives have been claimed [1]. Some of the most common classes of antibiotics administered to dairy cows are aminoglycosides, tetracyclines and fluoroquinolones [2]. Antibiotics act by penetrating the bacterial cells, altering its overall permeability and resulting in cell lysis.

Although antibiotics have significantly reduced disease occurrences and increased feed efficiency, their widespread use has raised serious public health concerns over the years [3]. In order to cope with increasing milk demand, practices of antibiotic administration to cows in the form of IV injections and regular feed additives has led to its over-use and misuse over the years. These pharmacologically active metabolites, known as “residues”, accumulate in the body of the animal over time [4]. That is to say, when a drug is administered to the cow, it is broken down by the body. Most parts of the antibiotics get absorbed into the bloodstream (bioavailability), while the rest is excreted in the form of urine or feces, depending upon the animal itself and the dose provided. However, constant exposure over time results in the animal being antibiotic resistant, thus requiring higher doses to overcome the illness [5]. This can reflect in the antibiotics being present in animal products such as milk, eggs and meat, which becomes a consequential public health concern [6,7].

Milk is a versatile and wholesome food that has been established as a great source of essential nutrients for centuries. It is fortified with appropriate amounts of fat, protein and vitamins, and it is extensively consumed as itself or its by-products [8]. In the year 2019, 552 MMT (million metric tons) of bovine milk was produced worldwide, of which India was the largest consumer at 77.4 MMT [9]. Being so widely consumed, it becomes imperative to monitor the amount of antibiotics present in milk before it reaches the consumer. To ensure public safety, the International Food Standard (Codex Alimentarius), in collaboration with Food and Agricultural Organization (FAO) and the World Health Organization (WHO), has set strict guidelines and threshold limits for the presence of antibiotic residues in milk [10].

Gentamicin is one such aminoglycosidic antibiotic used in the treatment of severe bacterial infections such as mastitis and metritis. It has broad spectrum activity against pathogens including *Escherichia. Coli*, *Streptococcus*, *Staphylococcus* and many others [11]. Derived from *Micromonospora purpurea,* gentamicin sulphate is made up of four major units- C_1_, C_1a_, C_2_ and C_2a_ and one minor component C_2b_ [12].While most of the gentamicin is renally excreted, intramammary administration tends to show residues in milk samples varying from 78 to 256 h from treatment time [13]. Toxicological studies of gentamicin reveal possible ototoxicity and nephrotoxicity among vulnerable groups on oral consumption [14]. Therefore, to overcome the above-mentioned challenges, the focus of this presented work was to design and develop a quick and reliable detection system to determine levels of antibiotics such as gentamicin in milk.

Liquid chromatography-mass spectroscopy (LC-MS) has been considered the “gold standard” for the detection of antibiotics in milk. However, its long process time and analysis cost per sample has had researchers looking for a cheaper and more effective alternative [15]. With the advent of molecular technique such as antibodies and DNA, biosensors have garnered acclaim for their quick response time and sensitivity [16]. This study used DNA aptamers, which are short sequences <100 base pairs long, with the ability to bind specifically to the molecule of interest [17]. The respective oligonucleotides are selected using the SELEX process (systematic evolution of ligands by exponential enrichment) depending on their dissociation constant (K_d_). The oligos that bind were eluted after several screening rounds and polymerase chain reaction (PCR) was used to make multiple copies [18].

Herein, a paper-based sensor for the easy detection of gentamicin in milk samples has been fabricated and developed. The localized surface plasmon resonance (LSPR) property of gold nanoparticles in combination with the specificity of aptamers has been used in a colorimetric assay [19]. Aptamer-coated gold nanoparticles demonstrated a strong absorbance peak λ_max_ = 520 nm due to the excitation of plasmons. When various concentrations of gentamicin were introduced, the affinity of the aptamers increased towards gentamicin, leaving the AuNPs bear and susceptible to salt-induced color change from red to blue. Similar noteworthy studies using gold nanoparticles have been conducted in the detection of contaminants [20] and antibiotics [21,22] in milk. However, this application of a paper substrate aims at the feasibility of having an on-farm, cost- effective, point- of-care device for screening of antibiotics. This device would also help veterinarians and farmers to make mindful decisions on administering antibiotics to cows. Furthermore, to evaluate its deployability and the extent of color development, spectroscopic and camera-based image processing techniques were performed on this sensor.

## 2. Materials and Methods

### 2.1. Materials

Tetrachloroaurate (III) hydrate (HAuCl_4_·3H_2_O), Tris, EDTA, sodium citrate and nitrocellulose membranes (Whatman^®^ Protran^®^) were sourced from Millipore Sigma (Oakville, ON, Canada). The DNA aptamers sequence was synthesized and acquired form IDT Technologies (https://idtdna.com, Coralville, IA, USA). The sequence used for the analysis (GA) was 5′- GGG ACT TGG TTT AGG TAA TGA GTC CC- 3′, which was referenced from Rowe et al. [23]. The aptamers were acquired in a lyophilized form using 1X Tris-EDTA (TE) buffer. To play the role of interfering molecules, BSA, D-fructose and β-lactose, commonly found in milk, were purchased from Millipore Sigma (Oakville, ON, Canada). ciprofloxacin hydrochloride monohydrate (C_17_H_18_FN_3_O_3_·HCl·H_2_O) was sourced from LKT Labs, while amoxicillin (C_16_H_25_N_3_O_8_S) was purchased from Fisher Scientific (Mississauga, ON, USA). TE buffer was prepared freshly whenever required using Milli- Q water (18.2 MΩ, DI water).

### 2.2. Gold Nanoparticle Synthesis and Characterization

Gold nanoparticles were prepared by a single step citrate reduction method. All glassware and stir-bars were cleaned thoroughly and oven dried before use. In a typical synthesis, 1 mM of HAuCl_4_ was dissolved in MilliQ water and constantly stirred while it was brought to a rolling boil. Next, 38.8 mM trisodium citrate dihydrate was rapidly added to the boiling mixture. A significant color change from pale yellow to light blue to wine red was observed. The heat was turned off and allowed to stir for 2 h until the colloidal gold sol reached room temperature. The prepared AuNPs were stored for later use, in the dark at 4 °C [24]. Spectral analysis of the synthesized AuNPs were performed using Synergy H1 Multimodal Plate reader (Biotek^®^ Instruments Inc., Winooski, VT, USA). The size and structure of the nanoparticles were analyzed using transmission electron microscope (FEI Tecnai G2 F20, San Francisco, CA, USA).

### 2.3. Preparation of Aptamer and Modified AuNPs

The aptamers were received in the form of a dry pellet. During the resuspension procedure, the aptamer vial was first centrifuged at 10,000 rpm for 30 s. To obtain a total stock concentration of 100 µM, 8 mL of Tris-EDTA buffer (10 mM Tris, 0.1 mM EDTA, pH 7.5) was prepared. This solution was briefly heated in a double boiler set up to uncoil the DNA oligos at 70 °C. It was allowed to cool back to room temperature for 20 min and later stored at −20 °C for further experiments. The stock solution was diluted to 1 µM working solution maintained at pH7.4 for all consecutive experimentation. A 96-well plate setup for full spectral analyses were used to as proof of concept. All optimizations were first conducted in a 96-well plate format and further applied on a paper substrate. Fresh dilutions of gentamicin were prepared in 1X TE pH 7.4, ranging from 3 µM to 1 nM from 10 µM stock solutions. The absorbance ratio of A640/A520 was calculated to plot the standard curve for the sensor and derive its sensitivity.

### 2.4. Preparation and Detection of Gentamicin on Paper-Substrate

**Figure 1:**
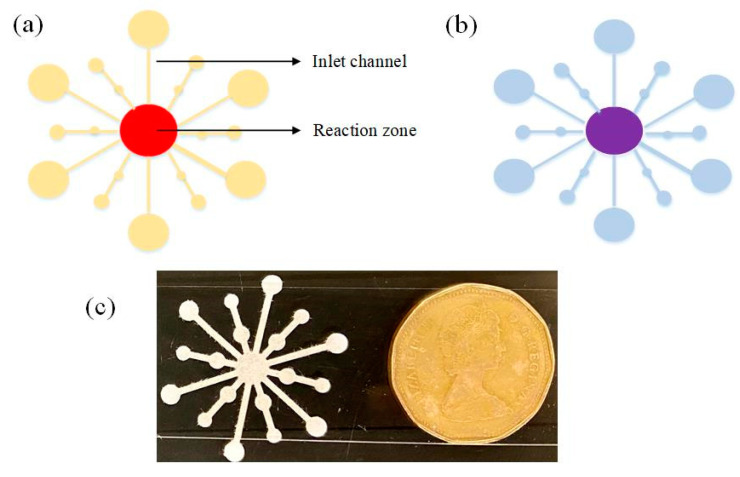
Schematic of the microfluidic paper biosensor depicting channels and reaction zones (**a**) color of reaction zone before the introduction of gentamicin and (**b**) color change from red to purple in the reaction zone after interaction. (**c**) The paper biosensor the size of a dollar coin.

The paper-based microfluidic device was cut out of a nitrocellulose membrane section. Nitrocellulose membrane (NC) was chosen as substrate for its uniform pore size 0.45 µm and unreactive property to a wide range of immobilized proteins and DNA strands [25]. Additionally, the NC surface is smoother in comparison to other paper substrates, contributing to better flow characteristics and higher stability. Figure 1a,b schematically represents the paper substrate before and after the reaction. The paper device was prepared by a flower shaped punching instrument (McGill^®^ 64512 Paper Blossoms Lever Punch) made of stainless steel. The mold was first cleaned with 70% isopropyl alcohol to remove dust/dirt deposits before punching. Post punching, the flowers were placed in a clean dry cabinet until further use. The dimensions were 1.25 × 1 inches comprised of 6 large arm channels and 6 small arm channels connected to a central area (reaction zone). The larger arm channels, which were used to load the sample, were 1.5 mm and the reaction zone had a diameter of 4 mm. In a typical experiment, 10 µL of synthesized AuNP was added to 1 µL (1 µM) aptamer solution, which was added to a clean microfuge tube. The solutions were allowed to bind for 15 min with mild shaking at room temperature. Next, 11 µL of the prepared mixture was added to paper substrate and air dried for 10 min. Several similar paper devices were prepared and stored in a clean, dry atmosphere (away from sunlight and corrosive fumes) up to 30 days for further use. Next, 5 µL of the desired concentration of gentamicin was added to the inlet arms and allowed to dry, after which, 5 µL of NaCl was added. The color change was monitored after the reaction was complete, marked by the drying of the reaction zone.

### 2.5. Real Sample Detection of Gentamicin

To explore the practical applicability of the colorimetric and paper sensor, skimmed milk (2%) spiked with gentamicin was used. Milk, being a complex matrix of proteins and caseins was first pretreated to remove these interfering materials described in previous studies [26]. In a typical experiment, 1 mL of milk sample was spiked with various concentrations on gentamicin before the pretreatment process. To this, 1 M HCl (pH 4.5) was added to precipitate the caseins, followed by centrifugation at 12,000 rpm for 5 min. The supernatant was separated and transferred to a fresh centrifuge tube, to which 300 µL methanol was added and the centrifugation process was repeated. The final clear supernatant was allowed to interact with ssDNA modified gold nanoparticles in the ratio 1:10. Spectral sweep data were obtained from 200–700 nm and the peak absorbance ratio of A640/A520 was calculated.

### 2.6. Imaging and Analysis of Paper Sensor

To estimate the LOD on the modified paper device, gentamicin-spiked buffer samples and milk samples were used. The paper experiment was repeated at least 3 times following the protocol illustrated in Section 2.4. The camera-based LOD for the paper sensor used the images captured on the 12 MP rear camera of iPhone 11, using a white background. RGB color analysis was performed on the obtained images (without modification) using Image J software 1.8 [27]. The images were loaded and split into individual channels of red (R), green (G) and blue (B). The reaction zone was analyzed for its red and blue intensities for both spiked TE and milk samples. The color intensity of both sample sets were plotted as a function of B/R vs concentration(nM), where B and R were blue and red intensities per pixel area of the reaction zone. The camera- based LOD was estimated using formula 3(standard deviation)/slope [28].

## 3. Results

### 3.1. Gold Nanoparticles in Optical Detection

**Figure 2:**
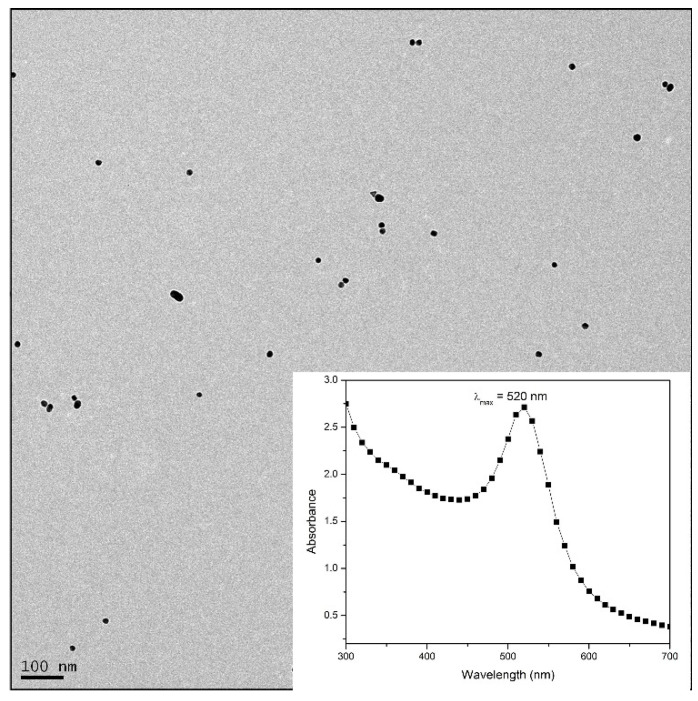
TEM of Au nanoparticles with the inset showing UV-Vis spectra of as-prepared AuNPs.

Metal nanoparticles, especially gold nanoparticles, have been applied in the field of optical biosensing in the past couple of decades. The most extensively explored property of gold nanoparticles is the property of localized surface plasmon resonance (LSPR) [29]. The optical property of metal nanostructures originates from its interaction with an incident light beam. This interaction causes collective oscillations/vibrations in the electron cloud of the nanoparticle, giving rise to the phenomenon of LSPR. Noble metals, such as gold and silver nanoparticles, are well known to exhibit unique SPR bands and hence play a pivotal role in the colorimetric detection of biological substances [30]. LSPR is influenced greatly by both absorption and scattering properties and these optical phenomena have contributed to the simplest form of biosensing. The proposed paper-based biosensor is an example of an aggregation sensor, which results in an immediate color change on changes in ionic strength or pH [19]. Here, AuNPs were prepared using a bottom-up technique (detailed in Section 2.2) and visualized under the TEM as shown in Figure 2. The TEM revealed homogeneous spherical morphology with an average size of ~15 nm (calculated using Image J). They were later subjected to Vis spectroscopy, where maximum absorbance of 520 nm was observed when the nanoparticles were subjected to wavelengths varying from 200–700 nm (inset).

**Figure 3:**
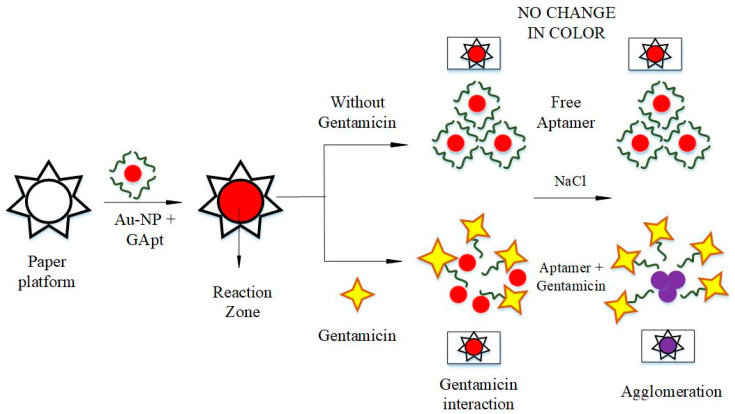
Schematic representation of the working of the prepared paper biosensor, which uses a colorimetric detection approach for gentamicin.

A schematic representation of the technique used for the detection of gentamicin on the paper substrate is illustrated in Figure 3. Aptamers added to the gold nanoparticle suspension remained free in the absence of gentamicin, while shielding the interfering NaCl. The overall resulting color remained deep-pink indicating no color change. On the other hand, a color change to purple was observed when the gentamicin-specific aptamer interacted with the gentamicin present in the sample.

### 3.2. Optimization of Sensing Parameters

**Figure 4:**
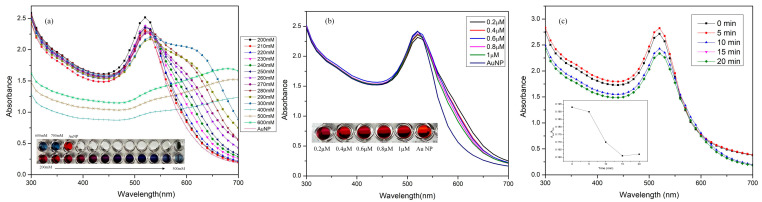
Optimization of: (**a**) NaCl concentration varied from 200 mM to 600 mM. Inset: the 96-well plate experiment depicting the extent of AuNP agglomeration and color gradation from deep red to purple to blue. (**b**) Aptamer concentration varied from 0.2–1 µM against 280 mM NaCl. (**c**) Interaction time between the aptamer-coated AuNP incubated for various lengths time and (inset) A640/A520 absorbance ratio vs incubation time.

The following experiments were performed to optimize the functioning of the label-free colorimetric detection of gentamicin: (i) NaCl concentration, (ii) aptamer concentration and (iii) aptamer interaction time. As introduced earlier, sodium ions are known to disrupt the ionic stability of the prepared AuNP, resulting in agglomeration. Hence, to optimize sodium ion concentration for AuNP aggregation, 20 µL aliquots of NaCl with concentrations varying from 200–600 mM were introduced to 200 µL of prepared AuNP solution in a microplate well. Spectroscopic study of the resultant AuNP particles showed no size changes until 100 mM, while increasing concentration of NaCl (Figure 4a and inset) revealed visible color change from dark-red to purple until 280 mM. Higher concentrations (300 mM and above) demonstrated noteworthy sedimentation of AuNP, resulting in the solution to turn grey-ish. Hence, for the successful detection of gentamicin, the concentration of NaCl was carefully selected to be 280 mM.

Next, the concentration of gentamicin aptamer (GA) was optimized by introducing various concentrations ranging from 0.2–1 µM. The ssDNA aptamer (GA) undergoes significant conformational changes to effectively bind to gentamicin [31]. The idea of optimizing aptamer concentration was to estimate the shielding ability of the AuNPs modified by GA against NaCl. Spectral data of the interaction between different aliquots of aptamer spiked to 200 µL of AuNPs in the microplate, followed by the addition of 280 mM NaCl, has been shown in Figure 4b and inset. The absorbance ratio A640/A520 was calculated to be the highest for 0.2 µM aptamer and least for 1 µM concentration. The concentration of the aptamer required for the assay was chosen such that the aptamers were evenly distributed over the AuNP surface and did not cause steric hindrance. Hence, 1 µM aptamer concentration was chosen for further experiments. Lastly, GA was allowed to interact with 200 µL of AuNP and incubated for different time periods, before 280 mM NaCl was added to it [32]. The absorbance ratio A640/A520 indicated that increasing interaction time increased the shielding effect of NaCl but had no effect after the 15 min mark (Figure 4c and inset). Hence the average interaction time between the aptamer and gold nanoparticles were maintained at 15 min for all further experiments. After each addition step, intermittent shaking and incubating in dark was performed to avoid interaction of light with the sample. Finally, 200 µL of AuNPs modified with 20 µL of 1 µM GA and 20 µL of 280 mM NaCl were used in all Vis spectroscopic studies.

## 4. Discussion

### 4.1. Sensor Validation

**Figure 5:**
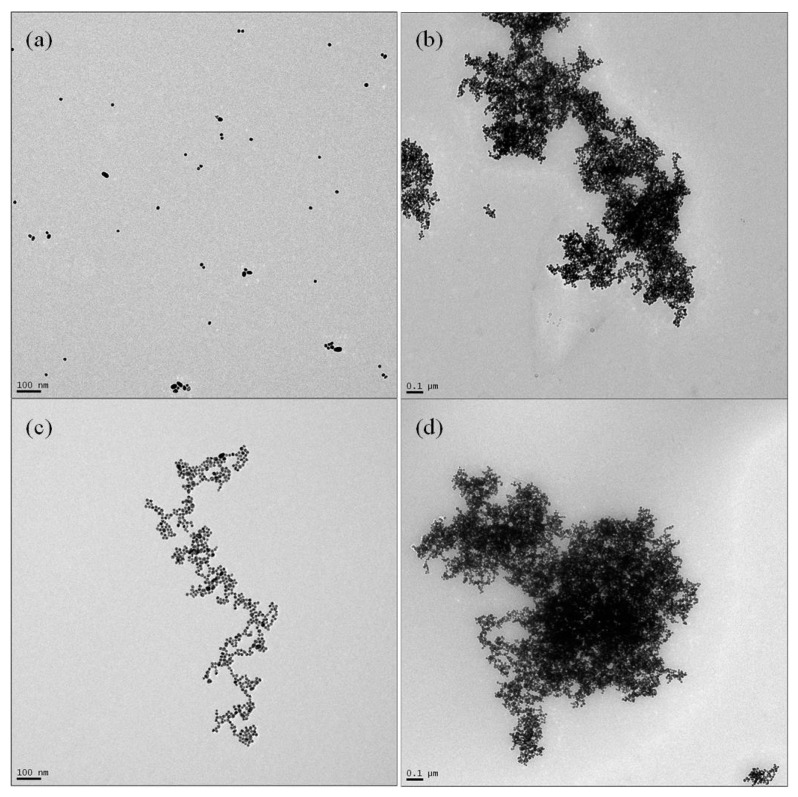
TEM images of the following: (**a**) untreated AuNP, (**b**) AuNP + 280 mM NaCl, (**c**) AuNP + 1 µM GA + 280 mM NaCl, (**d**) AuNP + 1 µM GA + 1500 nM gentamicin + 280 mM NaCl.

Morphological evidence to validate the working of the biosensor was performed in tandem with spectroscopic studies. Through the TEM images, the extent of aggregation of AuNP on the addition of NaCl and gentamicin was visualized. Presented in the results of Figure 5, four scenarios were analyzed: (A) untreated AuNPs, (B) AuNP + 280 mM NaCl, (C) AuNP incubated with GA + NaCl and (D) AuNP incubated with 1 µM GA followed by the addition of a higher chosen concentration (1500 nM) gentamicin and 280 mM NaCl. The untreated nanoparticles were visualized as homogeneously spaced spherical structures without any agglomeration. Ideally, the surface of gold nanoparticles is coated with capping agents to avoid clumping of AuNPs, but no such capping agents were used in this research. The prepared AuNPs were stored in a tinted container for 60 days at 4 °C and analyzed under the TEM. No agglomeration/flocculation was noted, as shown in Figure 5a. The addition of 280 mM NaCl caused the agglomeration of AuNPs, suggesting the action of varying surface charge. The change in ionic strength of the solution varied the morphology of AuNPs significantly, promoting the interaction of the energy barriers between the AuNP (Figure 5b). Here, conduction electrons between two adjacent particles were delocalized, causing them to share amongst themselves, resulting in a red shift occurring and the LSPR phenomenon tuning down to lower energies. The absorbance spectroscopic technique picked up the inherent changes in λ_max_ as a consequence of particle destabilization and the appearance of new peaks at longer wavelengths due to red shift. Figure 5c shows the morphological change undergone by AuNPs on the addition of 1 µM GA after an incubation period of 15 min, followed by the addition of NaCl. The ssDNA aptamer interacted through noncovalent bonding with the AuNPs via the available free nitrogen groups on its surface. This interaction is sufficient to anchor the aptamer to AuNPs, increasing its stability by repelling NaCl. In addition to TEM data, Vis spectroscopic data revealed comparable results to unmodified AuNP, further validating the extent of aggregation. The final leg of the experiment was the addition of a known concentration of gentamicin to the solution containing aptamer modified AuNP, completed by the addition of NaCl. Figure 5d shows the extent of agglomeration of AuNP on the interaction of 1500 nM of gentamicin to the aptamer solution, which provided a violet color change. The absorbance spectra showed the formation of a secondary significant peak at λ = 640 nm, corresponding to a red shift.

**Figure 6:**
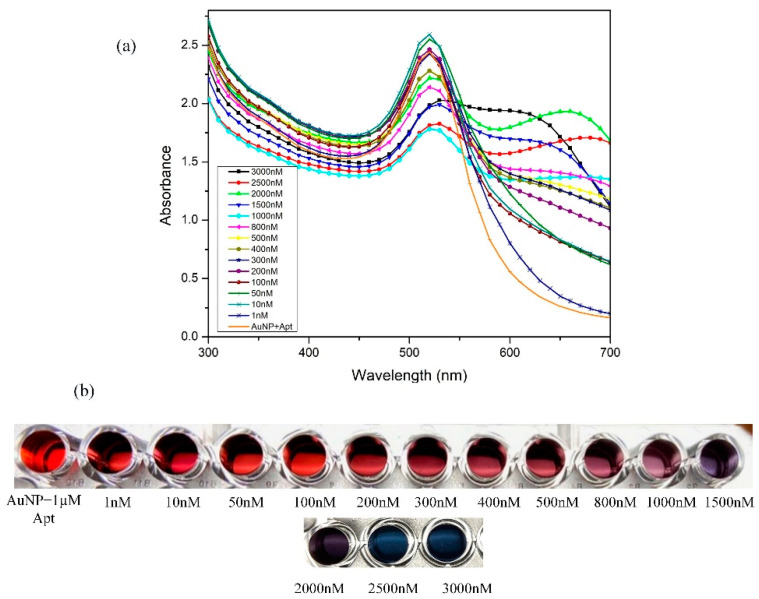
(**a**) Spectral data of different conditions on a 96-well plate setup: AuNP, AuNP + Aptamer and interactions with various concentration of gentamicin, and (**b**) an image of a trial experiment performed on a 96-well plate and representative concentrations labelled using 200 µL AuNP + 1 µM GA + x nM gentamicin + 280 mM NaCl.

Varying concentrations of gentamicin from 0–3000 nM were examined spectroscopically using a microplate (Figure 6a) and its corresponding color change is depicted in Figure 6b.

**Figure 7:**
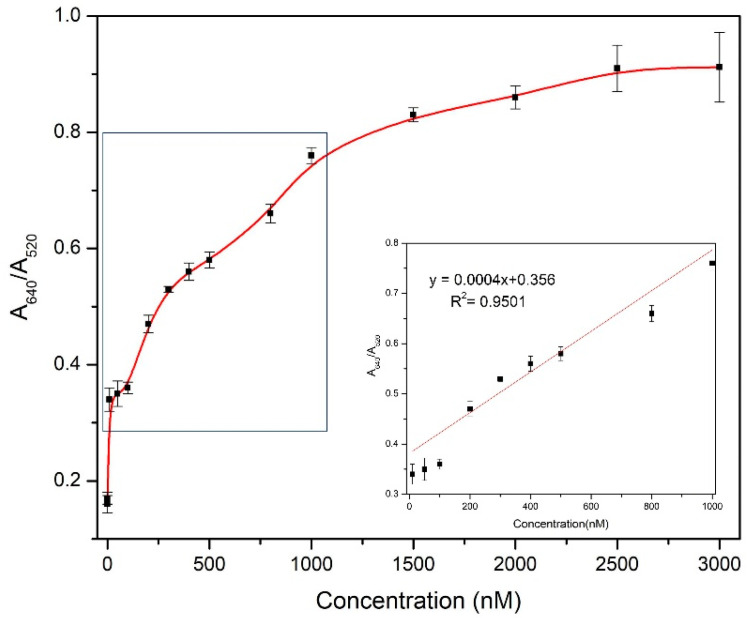
Calibration graph obtained from calculating the ratios of absorbance A640/A520 against increasing concentration of gentamicin (0–3000 nM). The inset shown the linear range of the biosensor, 10–1000 nM, with R^2^ = 0.95.

The next step to the experiment was to determine the limit of detection (LOD) of the prepared gentamicin biosensor. A significant color gradation from wine-red (unmodified/blank) AuNPs to deep purple to blue was observed with increasing concentrations of gentamicin. An evident reduction in peak absorbance at 520 nm was witnessed, while a new peak at 640 nm emerged on the addition of gentamicin. The observed data were first plotted as a full range concentration curve by calculating the ratio of absorbance between A640/A520. The ratio of the absorbance was plotted against the concentration of gentamicin, as shown in Figure 7. Linearity was observed from 10–1000 nM following the equation y = 0.0004x + 0.356, with R^2^ = 0.95 Figure 7 (inset). The limit of detection (LOD) was determined using standard deviation method with LOD = 3σ/S, where σ is the standard deviation of the y-intercept and the S represents the slope of the standard graph. The error bars were obtained from performing trials (n = 3) to confirm obtained results. The limit of detection of this sensor was calculated to be 225 nM.

**Figure 8:**
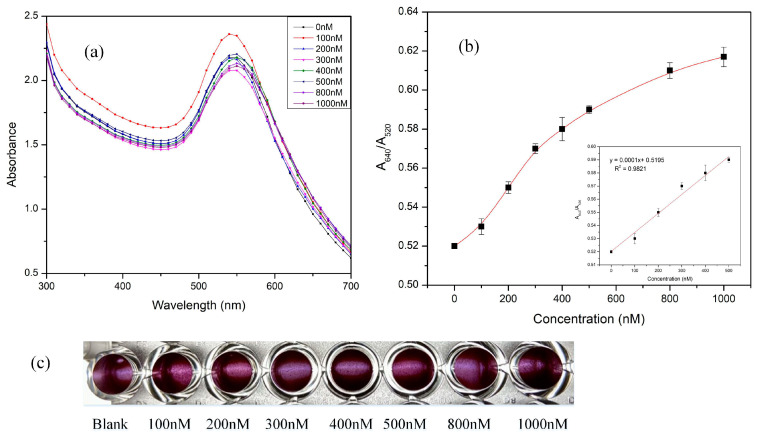
(**a**) Shown is the spectral data of preconditioned gentamicin-spiked milk samples on interaction with gentamicin aptamer (200 µL AuNP + 1 µM GA + x nM (20 µL) gentamicin spiked milk + 280 mM NaCl). (**b**) A640/A520 ratios of the spectral data and inset shows the linear range of the data (**c**) experiments performed on processed milk samples using a 96-well plate.

Further, skimmed milk samples, spiked with gentamicin were first conditioned before assay as detailed in Section 2.6. Visible spectral data depicted in Figure 8a were used to confirm the LSPR response of the AuNPs by repeating the same experiment on a 96-well plate. Concentrations concurring with the linear range of the TE buffer spiked gentamicin samples were chosen for the milk study. The color varied from gentamicin samples in buffer due to the extraction technique performed and as a consequence of pH variation (pH 7.4 for TE buffer samples and pH 5.2 for extracted milk samples). Hence, to understand the gradation better, a calibration graph was plotted, (Figure 8b and inset) for the absorbance ratios of A640/A520 against concentrations ranging from 0.52–0.61. The linear range of the absorbance ratios for concentrations ranging from (0–500 nM) followed the equation y = 0.0001x + 0.519 (R^2^ = 0.9821). Furthermore, increasing gentamicin concentrations revealed visible precipitation and sedimentation of AuNP, as shown in a 96-well plate experiment in Figure 8c. The LOD for gentamicin spiked samples using Vis-spec studies was calculated to be 210 nM.

### 4.2. Gentamicin Paper Assay

**Figure 9:**
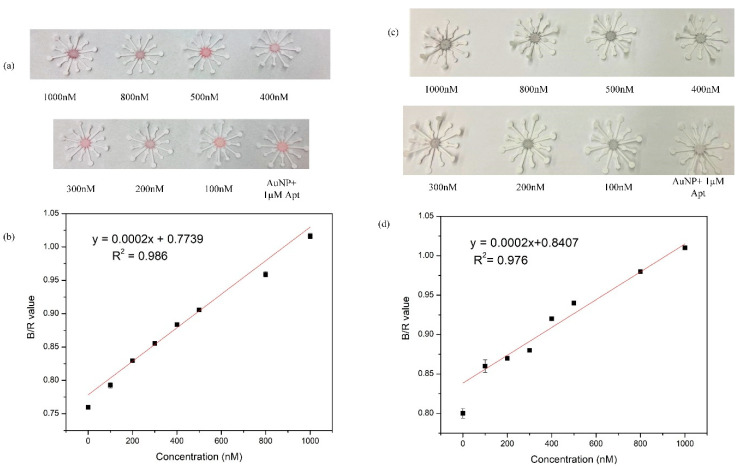
(**a**) Various concentrations of gentamicin samples in TE buffer (100–1000 nM) detected on paper substrate. (**b**) B/R values obtained using Image J plotted against various concentrations of gentamicin. (**c**) Milk samples spiked with known concentrations of gentamicin have been analyzed (100–1000 nM) on paper. (**d**) Corresponding B/R values (milk samples) vs concentration.

The paper sensor application is an extension to the colorimetric sensing, preformed on the 96-well plate. The paper sensor design uses a very simple fabrication technique on chromatographic paper. Of the four wider arms, two were used for the addition of reactants NaCl and gentamicin, while a solution of AuNP and gentamicin was added to the reaction zone. The shorter arms would act as absorbent pads for excess fluid applied to the larger/wider arms. Capillary action allows the sample to flow to the center of the paper flower. The quantity of reactants used on the paper substrate varied from that of the microplate experiment, but the concentration was maintained as optimized, detailed in Section 2.4. Red and blue channel intensities for both TE spiked samples and milk samples plotted as a function of B/R vs gentamicin concentration. The B/R values for TE gentamicin samples yielded a linear range from 0–1000 nM following the equation y = 0.0002x + 0.7739 (R^2^ = 0.986) (Figure 9a,b). The LOD was calculated to be 150 nM. Similarly, B/R values for gentamicin-spiked milk samples (Figure 9c,d) followed the linear equation y = 0.0002x + 0.8407 (R^2^ = 0.976) resulting in an LOD of 300 nM. The time taken for the reactants to combine by capillary effect and dry up to produce a color change was recorded to be 2 min.

### 4.3. Selectivity Studies

**Figure 10:**
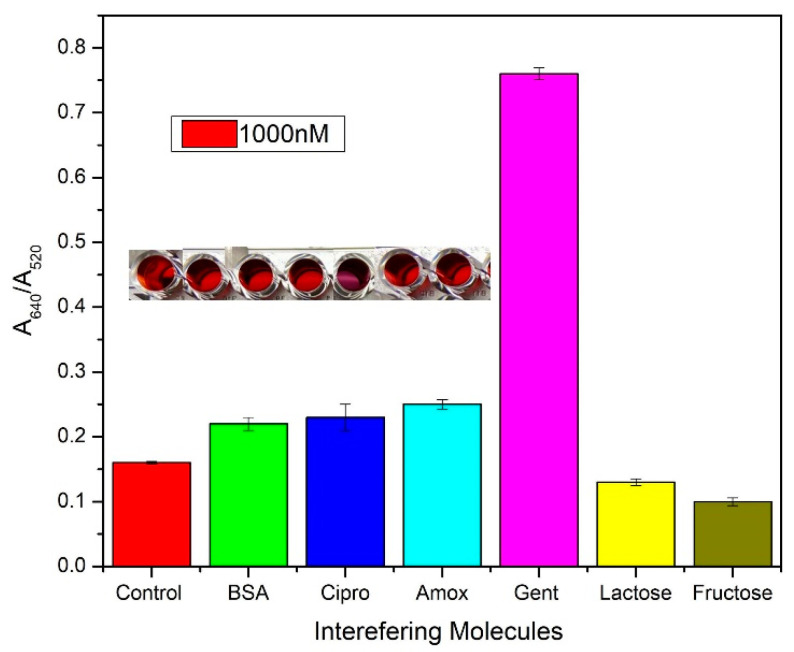
Interference studies performed with different molecules—bovine serum albumin (BSA), ciprofloxacin, amoxicillin, gentamicin, lactose and fructose by adding 200 µL AuNP + 1 µM GA + 1000 nM of interfering molecule in TE buffer (pH 7.4) + 280 mM NaCl. Inset: photograph of the well plate showing color change in the order of interfering molecules.

Selectivity studies are an important point to consider while designing a sensor. In practical applications where several molecules might cause the occurrence of a false positive/negative, a selective analysis becomes key. The possibility of the aptamer not interacting with a molecule other than gentamicin was established in the SELEX process, however, in a sensor application several number of factors could contribute to an incorrect response. Hence, various possible interfering molecules present in milk or that otherwise have a comparable structure/molecular weight were tested against our modified paper sensor. In total, 1000 nM concentration of all interfering molecules were introduced to the paper sensor and the spectroscopic data were obtained. The absorbance ratio of A640/A520 was plot to conclude a remarkable response for the gentamicin but not for any of the interfering molecules Figure 10. Similar antibiotics such as amoxicillin and ciprofloxacin were tested against the gentamicin aptasensor and an A640/A520 value close to control was observed, confirming no cross-reactivity.

## 5. Conclusions

The detection of antibiotics in milk is a serious concern in the field of agriculture. Despite measures to carefully analyze antibiotic levels, there still remains a need to determine gentamicin levels on farm. Keeping this in mind, the paper sensor is an effort to reduce the assay time required to determine a “yes/no” check before the milking process. This will, in turn, reduce the overall economic losses associated with discarding batches of milk containing high levels of gentamicin at the collection area. The design of the paper sensor took into consideration the International Food standard CODEX Alimentarius Maximum Residues Limit (MRLs) (200 µg/L, 418 nM), according to which, the sensitivity of the aptamer paper sensor using milk and spiked TE samples fall within permissible range. One of the major advantages associated with sensor usage is not requiring high-end spectroscopic techniques or reagents for qualitatively determining the presence or absence of gentamicin. A disadvantage of the assay could be: (i) possible cross-contamination between samples and (ii) the addition of too much sample into the channels in such a way that it floods into the detection area, thus clogging it or (iii) improper handling and storage of modified paper flowers between uses. However, this research is a step towards fabricating a point-of-care device that can be deployed towards ensuring food security.

## Data Availability

Data sharing not applicable.
